# Modern Management of Pregnancy in Systemic Lupus Erythematosus: From Prenatal Counseling to Postpartum Support

**DOI:** 10.3390/jcm13123454

**Published:** 2024-06-13

**Authors:** Anna Gamba, Margherita Zen, Roberto Depascale, Antonia Calligaro, Mariele Gatto, Luca Iaccarino, Andrea Doria

**Affiliations:** 1 Rheumatology Unit, Department of Medicine-DIMED, University of Padova, 35128 Padova, Italy; 2 Academic Rheumatology Centre, Department of Clinical and Biological Sciences, University of Turin, AO Mauriziano di Torino, 10128 Turin, Italy

**Keywords:** systemic lupus erythematosus, preconception management, disease monitoring, adverse pregnancy outcomes, therapeutic strategies

## Abstract

Systemic lupus erythematosus (SLE) is a chronic autoimmune disease that predominantly affects women of childbearing age. Pregnancy in SLE patients poses unique challenges due to the potential impact on maternal and fetal outcomes. We provide an overview of the management of SLE during pregnancy, including preconception risk stratification and counseling, treatment, and disease activity monitoring. These assessments are critical to minimize maternal and fetal adverse events in pregnant patients with SLE. Disease flares, preeclampsia, antiphospholipid syndrome complications, and maternal mortality are the major risks for a woman with SLE during gestation. Timely treatment of SLE relapse, differentiation of preeclampsia from lupus nephritis, and tailored management for antiphospholipid syndrome are essential for a successful pregnancy. Fetal outcomes include neonatal lupus (NL), preterm birth, cesarean delivery, fetal growth restriction (FGR), and small-for-gestational-age (SGA) infants. We focused on NL, linked to maternal anti-Ro/SS-A and anti-La/SS-B antibodies, which can lead to various manifestations, particularly cardiac abnormalities, in newborns. While there is a common consensus regarding the preventive effect of hydroxychloroquine, the role of echocardiographic monitoring and fluorinated steroid treatment is still debated. Finally, close postpartum monitoring and counseling for subsequent pregnancies are crucial aspects of care.

## 1. Introduction

Systemic lupus erythematosus (SLE) is a chronic autoimmune disease characterized by multiple organ involvements and a relapsing–remitting course. The cause is unknown; however, a number of genetic and environmental factors have been identified [1]. Like most autoimmune diseases, it predominantly affects females. The disease generally onsets in early adulthood, which means during fertile age. Historically, pregnancies in these patients were considered high risk due to the potential disease flare-ups and unfavorable maternal–fetal outcomes. Over time, more knowledge has been gained, leading to more frequent and safer pregnancies in patients with SLE. Nevertheless, compared to the general population, there are several factors that can negatively influence pregnancy in a woman with SLE, including disease activity, presence of some autoantibodies, pharmacological therapy, and comorbidities. Therefore, proper planning and competent management of pregnancy starting from the preconceptional period are required.

## 2. Counseling

Appropriate counseling is essential to address the multiple issues related to childbearing age. An integrated approach involving rheumatology and gynecology experts, while challenging to accomplish, can be highly beneficial for patients dealing with fertility and rheumatological issues. Furthermore, it is crucial that the specialist can effectively answer the questions and concerns that patients might have. Several studies [2] showed that the number of pregnancies in patients with systemic lupus erythematosus (SLE) is lower than in the general population. This discrepancy is not only due to disease activity and drugs, but it is also influenced by personal choices. These decisions often result from patients’ concerns about infertility, SLE activity during pregnancy, potential transmission of the disease to offspring, using harmful medications for the fetus, and worries that the disease might make them unable to take care of a child. Hence, it is imperative for rheumatologists to engage in counseling to alleviate the psychological stress associated with these issues.

### 2.1. Infertility

There is no clear evidence that SLE reduces female fertility. Nonetheless, some research [3] has found oophoritis and altered regulation of the hypothalamic–pituitary axis in SLE patients, while hyperprolactinemia is associated with disease flares. Furthermore, women with SLE have a reduced ovarian reserve, as indicated by their lower levels of anti-Müllerian hormone (AMH). Antiphospholipid and anti-prothrombin antibodies were reported at higher levels in patients with infertility compared to controls [4]. Additionally, there is a higher incidence of menstrual cycle irregularities, which make it more challenging to initiate pregnancy. Historically, a proportion of patients experienced infertility due to cyclophosphamide (CYC) use, leading to ovarian insufficiency. Over time, this risk has significantly decreased due to the combined use of CYC and a gonadotropin-releasing hormone analog (GnRHa), as well as the lower use of CYC due to the introduction of new therapeutic options for managing SLE, including mycophenolate mofetil (MMF). In situations where a woman’s fertility window is limited but the need to postpone pregnancy arises due to high disease activity and/or ongoing teratogenic therapy, the rheumatologist should refer the patient to a gynecologist. Indeed, there are several alternatives to natural conception, including oocyte cryopreservation or assisted reproductive techniques.

### 2.2. Contraception

Since planning a pregnancy is crucial for SLE patients, proper counseling on contraception is equally important. In the past, there was a belief that using combined estrogen–progestin pills (E-P) might trigger disease activity. However, several studies [5,6,7] have shown the safety profile of contraception with E-P pills and copper intrauterine devices (IUDs) in patients with inactive diseases, while the use of medicated IUDs should only be considered in specific cases where the benefits outweigh the thrombotic risk [8]. Nevertheless, 25% of patients taking teratogenic drugs do not use effective contraceptive methods [9]. Therefore, it is essential that rheumatologists reassure patients about the safety of contraceptive methods, emphasizing the need to delay gestation until disease control is achieved with pregnancy-compatible drugs. For patients with antiphospholipid antibodies (aPLs), with or without antiphospholipid syndrome (APS), or those with thrombotic risk factors independent from SLE, estrogen–progestin contraception is not recommended. In these cases, progestin-only pills or implants can be considered, carefully weighing the risks and benefits.

### 2.3. Preconception Risk Stratification

In women with SLE, several risk factors can lead to unfavorable pregnancy outcomes. The 2017 European League Against Rheumatism (EULAR) recommendations [7] suggested a helpful checklist of variables to consider during preconception counseling, as outlined and further detailed in Table 1. Additionally, factors applicable to the general population should be taken into consideration, including a woman’s age, diabetes mellitus, arterial hypertension, obesity, thyroid disorders, smoking/alcohol habits, and vaccination status [10]. Considering all these variables, pregnancy may be recommended, postponed, or contraindicated (Table 2).

Inactive disease for a minimum of 6–12 months before conception is essential to reduce the risk of adverse outcomes. The risk of an SLE flare during pregnancy is two-fold increased in patients with active disease prior to conception compared to those with inactive disease [11,12]. Relapse, in turn, increases the risk of fetal loss, preterm birth, preeclampsia (PE), and intrauterine growth restriction (IUGR). Most of flares occur in musculoskeletal, cutaneous, hematological, and renal systems. Notably, most disease flares during pregnancy affect the same organs involved before pregnancy [11,12]. For instance, if the disease was previously active in the skin, a recurrence of cutaneous manifestations is likely to occur more than other manifestations. Only renal involvement is a risk factor for both renal and non-renal flares.

### 2.4. Allowed Therapies during Pregnancy

In the preconception assessment, pharmacological therapy plays a pivotal role (Table 2). A number of studies and guidelines [12,13,14,15,16,17,18] advocate for the continuation or initiation of hydroxychloroquine (HCQ) during pregnancy. Indeed, it can contribute in maintaining disease control, and its discontinuation has been linked to an increased risk of flares during pregnancy [7]. The recommended dose is ≤400 mg/day, as a greater risk of fetal malformations above this dosage has been reported [18]. Other drugs with a favorable safety profile during pregnancy are oral glucocorticoids (OGCs), azathioprine (AZA), cyclosporine (CsA), and tacrolimus (TAK). These medications should be administered at the minimum effective dose, carefully considering the risk/benefit ratio associated with their use. Regarding OGCs, a study by Desai R. et al. [19] showed that high doses of OGCs (prednisone, PDN, >10 mg/day) are an independent risk factor for severe infections (defined as bacterial or opportunistic infections requiring hospitalization) in pregnant women with systemic inflammatory diseases, including SLE. OGCs are also associated with gestational hypertension, gestational diabetes, premature rupture of membranes, and small-for-gestational-age (SGA) newborns. Despite no evidence of a teratogenic effect, belimumab (BEL) and rituximab (RTX) are generally discontinued upon conception due to a lack of sufficient data regarding their safety profile during pregnancy. However, they may be continued in cases of severe disease despite the use of drugs that are considered safe during pregnancy. Specifically regarding BEL, EULAR and the British Society for Rheumatology guidelines suggest its prudent use in pregnancy [7,12], while the American College of Rheumatology (ACR) recommends its discontinuation [2]. However, the available data are gradually increasing. An interim analysis of the Belimumab Pregnancy Registry (BPR) was published in 2022, which showed no increased risk of birth defects in children of women exposed to BEL during pregnancy [20]. These results are in line with other limited case series in the literature [21,22,23,24]. In general, the limitations of these studies are the small sample size and a lack of comparison with pregnancies in patients with SLE not exposed to BEL. In fact, adverse events in pregnancy are more frequent in SLE than in the general population.

For the treatment of ongoing flares during pregnancy, the following treatment can be used, weighing risks and benefits: intravenous GCs, intravenous immunoglobulins (IVIGs), and plasma exchange. CYC can only be used in cases of severe organ failure or risk of maternal–fetal death. Use of antiplatelet and anticoagulant medications will be discussed later. Table 3 summarizes which SLE therapies can be used in pregnancy and which are contraindicated

### 2.5. Medications to Discontinue before Pregnancy

Drugs with demonstrated teratogenic effects should be discontinued before conception and replaced with pregnancy-safe alternatives. The effectiveness of not-harmful treatments in maintaining disease control should be assessed for at least 3–6 months before conception [2,7,25]. Timing of discontinuing medications depends on their half-life. Methotrexate should be stopped at least one month before conception, mycophenolate should be stopped at least 6 weeks before conception, and leflunomide should be halted when the pregnancy is planned with a washout period using cholestyramine. Beside immunosuppressants, some medications used in various SLE manifestations are contraindicated during pregnancy. ACE inhibitors/angiotensin receptor blockers (ARBs), prescribed for lupus nephritis as antihypertensive and antiproteinuric agents, should be discontinued due to the associated risk of fetal complications, including IUGR, cardiac and renal abnormalities, limb malformations, and oligohydramnios [26]. If necessary, alternative antihypertensive medications such as methyldopa, labetalol, and nifedipine can be administered. In APS patients on anticoagulant therapy, warfarin, due to its teratogenic effect, is replaced with low-molecular-weight heparin (LMWH) at therapeutic dosage. In managing arthritis and joint pain, NSAIDs can be used at the lowest effective dose until the 30th gestational week (GW), after which they are associated with an increased risk of ductus arteriosus constriction. 

### 2.6. Supportive Therapies during Pregnancy

Many studies [27,28,29,30,31] support vitamin D supplementation in pregnant women with SLE, emphasizing its anti-inflammatory and immunomodulatory effects. Additionally, a correlation has been observed between vitamin D deficiency and conditions such as PE and miscarriages. Moreover, vitamin D appears to play a protective role even in patients with aPLs. In vitro studies demonstrated a reduced expression of coagulation cascade activation molecules in endothelial cells exposed to anti-beta2-glycoproteinI antibodies (anti-β2GPI). Furthermore, an increased number of thrombotic events has been noted in cases of vitamin D deficiency. Its usage is crucial, especially in patients undergoing treatment with steroids or LMWH, considering their potential impact on reducing bone mass [7]. However, despite this evidence, not all pregnant patients routinely take vitamin D in clinical practice.

## 3. Pregnancy Follow-Up

**Figure 1:**
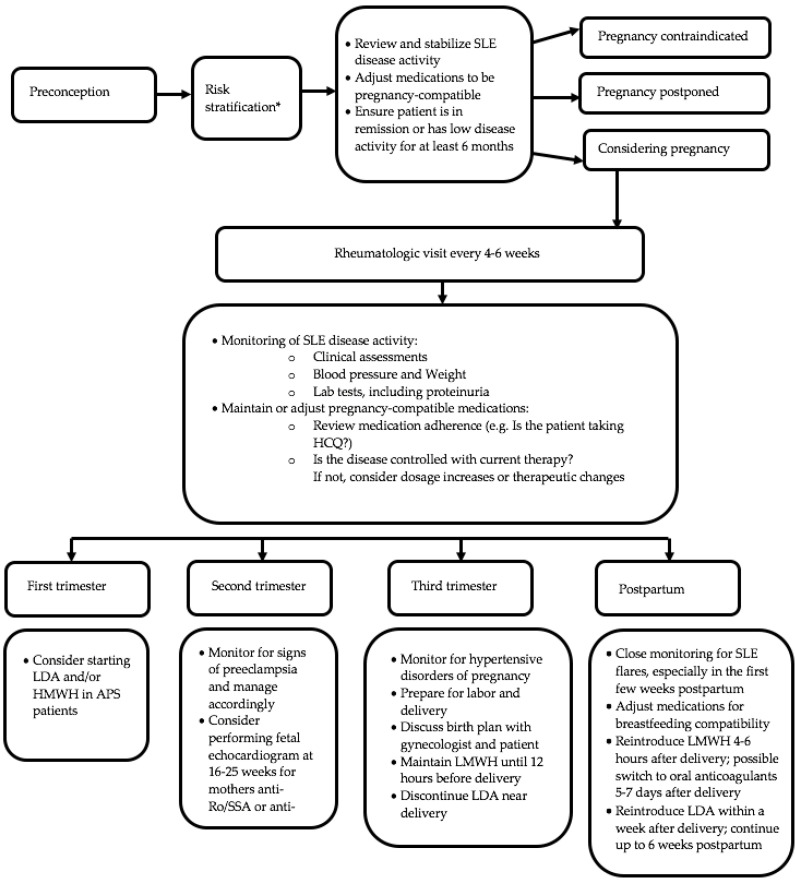
Pregnancy management in SLE patients. Legend: *: See Table 1 and Table 2; +: positive; APS: antiphospholipid syndrome; ASA HCQ: hydroxychloroquine; LMWH: low-molecular-weight heparin LDA: low-dose aspirin; SLE: systemic lupus erythematosus.

As observed, the prognosis of pregnancy largely depends on the disease activity, as flares are major risk factors for unfavorable maternal–fetal outcomes. Assessing disease activity during pregnancy can be challenging for rheumatologists, as there are clinical and laboratory changes in the gestational period that can mimic manifestations of SLE [32]. Indeed, pregnant women may present cutaneous features (facial rash, palmar rash, melasma), arthralgia, mild joint swelling, and postpartum alopecia. In order to ensure adequate blood flow to the placenta, there is a general vasodilation, resulting in increased hemodynamic load. This leads to hemodilution, causing mild anemia, mild thrombocytopenia, increased erythrocyte sedimentation rate (ESR), and an elevation in the glomerular filtration rate (GFR). Therefore, it is important to bear in mind that there could be a reduction in renal function with eGFR still within normal limits. As filtration increases, the excretion of proteins in urine also rises. Therefore, gestational proteinuria is considered within the normal range up to 500 mg/24 h. Pregnancy also triggers a prothrombotic condition linked to elevated coagulation factors I, VIII, IX, and X and reduced concentrations of anticoagulants like protein S and antithrombin. Normally, active SLE involves complement consumption. During pregnancy, there is an increase in complement values, especially the C3 fraction, the synthesis of which is stimulated by estrogen. In the PROMISSE study (Predictors of pRegnancy Outcome: bioMarker In antiphospholipid antibody Syndrome and Systemic lupus Erythematosus) [13], Buyon et al. analyzed 385 pregnancies in SLE patients. They found that a smaller increase in C3 in the second and third trimesters correlated with a higher risk of adverse pregnancy outcomes, defined as fetal death, neonatal death, preterm delivery due to hypertension, PE, placental insufficiency (PI), and SGA. Ideally, pregnancy in women with SLE should be managed by a multidisciplinary team, including a rheumatologist, gynecologist, obstetrician, and neonatologist. Regular assessments by the rheumatologist are crucial, involving close clinical and laboratory monitoring every 4–6 weeks to detect signs of disease activity early. Visits should include a physical examination, blood pressure assessment, hematological and biochemical biomarker evaluation (complete blood count, liver enzymes, creatinine, urine analysis, complement, anti-dsDNA titers), and use of disease activity indices. Over time, various clinimetric indices have been proposed for assessing disease during pregnancy such as the SLE-Pregnancy Disease Activity Index (SLEPDAI), the Lupus Activity Index (LAI) in Pregnancy (LAI-P), modified SLAM (m-SLAM) [33], modified-European consensus lupus activity measurement (m-ECLAM) [34,35], and the British Isles Lupus Assessment Group-2004 for pregnancy (BILAG2004-P) [36]. These indices have been modified from their original versions, excluding confounding factors between SLE and pregnancy [32]. Only LAI-P has been validated [37]. The characteristics of various clinimetric indices are provided in Table 4. In addition, the potential onset of gestational diabetes, especially in patients receiving steroid therapy, should be considered, and glucose tolerance tests, in agreement with local gynecological guidelines, should be performed. It is important to consider that a diabetogenic state commonly emerges as the pregnancy progresses into the third trimester. Monitoring patients with anti-SSA and anti-SSB antibodies will be addressed later. Figure 1 outlines the main assessments to perform in pregnancy management.

## 4. Adverse Pregnancy Outcomes 

A cross-sectional study compared pregnancy outcomes in 93,820 women diagnosed with SLE and 78,045,054 women without SLE admitted to hospitals in the United States between 1998 and 2015 [38]. Adverse events that were studied were maternal mortality, fetal mortality, PE, eclampsia, cesarean deliveries, non-delivery-related hospitalizations, and the duration of hospital stays. Over the 18-year study period, a declining trend in adverse events was observed in both groups, although it remained greater in the SLE group. Early fetal losses in the first trimester may be linked to common factors shared with the general population, such as chromosomal abnormalities, hormonal imbalances, and advanced maternal age. However, women with SLE face additional risk factors, including aPL/APS, disease activity (especially lupus nephritis), arterial hypertension, and organ damage. Beyond early adverse outcomes, the study identified a series of adverse pregnancy outcomes (APOs) occurring in the second and third trimesters, such as PE, eclampsia, IUGR, preterm birth, and SGA neonates. 

### 4.1. Maternal Outcomes

#### 4.1.1. Disease Flares and Renal Flares

One of the major risk factors for APO is the reactivation of SLE. Disease flares are, in turn, influenced by the control of SLE activity in the preconception period. The percentage of flares varies, ranging from 7 to 20% in women with inactive disease in the six months before pregnancy to 20 to 60% in those with active disease [39]. Smyth et al. conducted a meta-analysis of 37 studies, encompassing a total of 2751 pregnancies in women with SLE, including 1000 with LN. From this analysis, the most frequent maternal complications were disease flares, observed in a quarter of pregnancies, and active lupus nephritis, occurring in one out of six pregnancies [40]. Renal flares not only increase the risk of maternal complications, such as hypertension and PE (57% vs. 11%), but also elevate the likelihood of fetal complications, including preterm birth and fetal deaths (35% vs. 9%) [41,42].

Therefore, disease relapses, particularly renal ones, should be treated in a timely manner with aggressive immunosuppressive therapy. This may involve pulse intravenous non-fluorinated GCs (which do not cross the placenta), AZA, CNI, plasma exchange, and IVIGs. As the pregnancy progresses into the second trimester, careful consideration may be given to the use of cyclophosphamide, sharing with the patient the potential risks and benefits associated with this drug [43]. 

#### 4.1.2. Preeclampsia

Between 20% and 30% of SLE pregnancies are at risk of developing PE [38,44,45]. PE is defined as an elevation in blood pressure (>140/90 mmHg, measured on at least two occasions) accompanied by increased proteinuria. HELLP syndrome, characterized by thrombocytopenia, elevated liver enzymes, and hemolytic anemia, is a condition associated with PE. In cases where central nervous system manifestations, such as seizures, occur, it is referred to as eclampsia. Factors such as placental malformations, including altered remodeling of spiral arteries, genetic factors, and proinflammatory factors, especially dysregulation of the complement system, seem to contribute to its development. One of the significant challenges during pregnancy is distinguishing between a flare of LN and PE. Overall, disease activity is a risk factor for PE. Differential diagnosis is further complicated by the fact that LN itself is a risk factor for PE. Furthermore, a study has demonstrated that the use of PDN in the third trimester is associated with the development of PE [41]. The distinction is paramount because the treatment differs; LN is managed with immunosuppressants, whereas PE requires the delivery. Table 5 outlines the key biomarkers that, when considered together, contribute to an accurate differential diagnosis. From a clinical perspective, unlike PE, LN may present with signs of disease activity in other organs, such as skin, serous membranes, and the musculoskeletal system.

Maynard et al. [46] advocate for the performance of renal biopsies in cases where the differential diagnosis poses a challenge. They reference two studies, one from 2013 involving 197 pregnancy-related biopsies [47] and another from 2015 covering 11 pregnancies [48]. In the first cohort, significant complications (bleeding, fetal loss, infections, PI) occurred in only 3% of cases, while there were no major complications in the second cohort. Furthermore, in the second study, therapeutic decisions completely changed in 10 out of 11 cases based on the histological results of the biopsy. According to Maynard et al., the decision to perform a biopsy should be influenced by gestational age. Complications related to the procedure are minimal in the first trimester, but in the third trimester, it is more prudent to consider delaying the biopsy until postpartum due to the risk of an emergency preterm delivery. The greatest challenge is the second trimester, when the first-line approach might involve a personalized lupus therapy tailored to the patient’s characteristics, allowing pregnancy progression until a permissive gestational age for induced delivery.

Several studies showed that the early administration of low-dose aspirin (LDA) during pregnancy reduces the risk of PE. This drug is particularly recommended for patients with PE risk factors, including extreme age, primigravida status, arterial hypertension, pre-existing renal conditions, and aPLs [49]. As demonstrated in the ASPRE (Aspirin for Evidence-Based Preeclampsia Prevention) trial [50], initiating 150mg/day of LDA before the 16th GW reduces the risk of PE in high-risk women. The use of LDA is recommended by the EULAR group [7] and the American College of Obstetrics and Gynecology (ACOG) [51]. In both SLE and PE, tissue damage seems to be due to reactive oxygen species. Multiple studies [52,53,54] have indicated that HCQ reduces the synthesis of reactive oxygen species (ROS) and, consequently, their associated damage.

#### 4.1.3. Antiphospholipid Syndrome

The prevalence of aPLs in patients with SLE is 12–44% for anticardiolipin antibodies (aCL), 15–34% for lupus anticoagulant (LAC), and 10–19% for *anti*-β2GPI [55].

The presence of these antibodies is an independent risk factor for APO, including early (<10th GW) and late fetal deaths, thrombotic events, and PI. A number of in vitro and in vivo studies [56,57] have demonstrated the pathogenicity of these antibodies, which seem to target β2GPI, expressed by trophoblast cells, leading to tissue damage. However, the presence of aPLs alone is not sufficient for diagnosing APS. In 2023, ACR/EULAR [58] criteria for APS were established. These criteria require the concomitance of both clinical events and laboratory positivity for aPLs. APS is categorized into two main groups: thrombotic and obstetric APS. The former is characterized by macrovascular (arterial or venous), microvascular, cardiac valve, and hematological (thrombocytopenia) involvement. The latter is defined by the anamnestic presence of ≥3 pre-fetal deaths before the 10th GW; ≥3 early fetal deaths between the 10th and 15th GW; ≥1 fetal death (16th–33rd GW) without severe PE or severe PI; or severe PE and/or PI, associated or not with fetal death (Table 6). Laboratory criteria require the positivity of one or more aPLs, confirmed on at least one occasion at a minimum of 12 weeks apart from the first detection, within 3 years of a clinical event. It is important to point out that these are classification and not diagnostic criteria. When APS is diagnosed in the context of an SLE diagnosis, it is termed secondary; otherwise, it is considered primary (PAPS). Additionally, there are autoantibody profiles defined as “high-risk”: the presence of elevated LAC and triple positivity (LAC, aCL, and anti-β2GPI) are associated with worse outcomes. Addressing treatment, three situations should be distinguished: patients with aPL without a history of clinical events, patients with thrombotic APS, and patients with obstetric APS. According to the 2019 EULAR recommendations [59], pregnant patients with SLE and a high-risk aPL profile, without previous thrombotic or pregnancy episodes, should be treated with LDA (75–100 mg/day) during pregnancy. In pregnant patients with obstetric APS and SLE, LDA should be used from the preconception period, with the addition of prophylactic LMWH upon pregnancy confirmation. If the obstetric clinical event was a fetal death from the 10th to the 34th GW, the use of LMWH should be evaluated depending on the patient’s risk profile. Additionally, in patients with clinical events not sufficient to define obstetric APS, the use of LDA alone or in combination with LMWH should be taken into consideration. In patients with obstetric APS experiencing recurrent pregnancy complications despite prophylactic therapy with LDA and LMWH, escalation to therapeutic LMWH dosage or the addition of HCQ or low-dose PDN from the first trimester may be considered. In highly selective cases where conventional treatments have failed, IVIGs may be an option.

Pregnant women with SLE and thrombotic APS should be treated with a combination of LDA and therapeutic LMWH. LMWH should be maintained until 12 h before delivery and reintroduced 4-6 h after cesarean section or epidural catheter removal. If the patient has a high thromboembolic risk, LMWH can be converted to unfractionated heparin before delivery, as its effect is more rapidly reversible. LDA is discontinued near delivery, always considering the pregnant woman’s risk, and resumed within a week after delivery, continuing for up to 6 weeks postpartum due to the elevated thrombotic risk during the puerperium. In women at high risk, the LDA-LMWH combination can be continued until the onset of uterine contractions. Women who were on warfarin therapy before pregnancy can be transitioned to oral anticoagulants usually 5–7 days after delivery, in the absence of bleeding complications. Breastfeeding is compatible with both warfarin and LDA and LMWH.

#### 4.1.4. Maternal Mortality

There are not many recent data on maternal mortality. In a US national study of 13,555 SLE pregnancies from 2000 to 2003, Clowse et al. [60] found that the risk of maternal death (325/100,000 live births) was more than 20-fold higher than in the non-SLE population. A review by Ritchie J. et al. [61] reported 17 cases of maternal death. In all of them, there was active disease, and the most frequent causes were infection and flares of disease (40% and 30%, respectively). Other rarer causes were pulmonary embolus, pregnancy-associated cardiomyopathy, and adrenal failure due to abrupt glucocorticoid withdrawal.

### 4.2. Fetal Outcomes 

#### 4.2.1. Neonatal Lupus

NL is an autoimmune condition acquired passively by the newborn from mothers with anti-Ro/SS-A and anti-La/SS-B antibodies. Anti-SSA antibodies target the antigenic subunit of molecular weight 52 kD or 60 kD, known as anti-Ro52 and anti-Ro60, respectively. The former is more commonly found in cases of cardiac manifestations. Cardiac NL rarely occurs in offspring of women exclusively positive for anti-La/SS-B [62]. However, when both anti-Ro/SS-A and anti-La/SS-B antibodies are present, the likelihood of NL increases [63]. Buyon J. in 1993 [64] and Jaeggi E. in 2010 [65] showed that the lower the antibody titer, the lower the risk of newborn lupus. Overall, 40% of SLE patients have these antibodies, which are also found in other rheumatological conditions such as Sjogren’s syndrome, undifferentiated connective tissue diseases [66], and rheumatoid arthritis, as well as in the general population [67]. Notably, 50% of mothers of infants born with NL will develop the autoimmune disease after pregnancy [68]. Cutaneous manifestations of NL have also been identified in offspring of mothers who have only anti-U1RNP antibodies [69]. The precise pathogenetic mechanism of NL remains elusive. A cross-reaction between maternal autoantibodies and various embryonic tissues has been hypothesized. In fact, being IgG, the mother’s autoantibodies are able to cross the placenta from the second trimester. In vitro studies have demonstrated that anti-Ro/SS-A and anti-La/SS-B bind calcium-regulating molecules such as calcium channels T and L, which are present in cardiac conduction tissue. This binding would likely promote a local inflammatory response, leading to tissue damage. However, only 2% of offspring born to mothers carrying anti-SSA and anti-SSB antibodies develop manifestations of NL. Probably a genetic predisposition promotes progression to fibrosis or disrupts the inflammatory process. Some studies have indicated an increase in biomarkers of inflammation and cardiac distress (C reactive protein, metalloproteinases, NT-ProBNP) in the umbilical cord blood of neonates with cardiac manifestations compared to those without such manifestations [70]. A type of macrophage expresses high levels of sialic acid-binding Ig-like lecithin 1 (SIGLEC-1). It is a proinflammatory cell, upregulated by type I interferon (IFN). These macrophages were found in the cardiac tissue of fetuses with CHB [71]. A study conducted by Lisney et al. demonstrated a correlation where mothers of children with congenital heart block (CHB) exhibited significantly higher expression levels of SIGLEC-1 and IFN-α compared to mothers with healthy children [72]. The term “neonatal lupus” refers to a range of manifestations that can vary both in severity and duration. These manifestations include transient skin involvement, characterized by the appearance of annular erythematous lesions. Manifestations typically affect the face, scalp, and neck. These lesions may be present from birth or emerge between 4 and 6 weeks of life, usually auto-resolving in 17 weeks, when the mother’s autoantibodies disappear from the neonatal blood. Additionally, there may be transient and asymptomatic liver involvement, marked by a mild elevation in transaminases, as well as mild hepatosplenomegaly that can progress to cholestasis and hepatitis. Varying degrees of cytopenia can occur, occasionally progressing to aplastic anemia. Neurological involvement, often transient, may present with subtle symptoms and nonspecific neuroradiological signs, or with macrocephaly and hydrocephalus. However, the association with NL is not universally supported by all researchers. The most severe complication, but also the least frequent, involves cardiac involvement [69]. Cardiac NL occurs between the 18th and 26th GW, corresponding to the embryonic development period of cardiac tissue. About 2% of the newborns from mothers with anti-Ro/SS-A and anti-La/SS-B antibodies develop cardiac NL. The recurrence risk of cardiac NL in a subsequent pregnancy is 12–17%, whereas if the woman has already given birth to a child with non-cardiac NL manifestations, the likelihood is similar to that of patients with only anti-SSA and anti-SSB. Typically, it manifests without structural cardiac anomalies but with rhythm disturbances, notably atrioventricular congenital heart block (CHB) of the first, second, or third degree (complete CHB, CCHB). In the latter case, mortality reaches 17% by the 30th GW. Other potential cardiac complications include endocardial fibroelastosis and consequent dilatated cardiomyopathy, congestive heart failure, sinus bradycardia, valvular alterations, and myocarditis. Current recommendations suggest a close echocardiographic monitoring of the fetal heartbeat between the 18th and 26th GW. However, some studies [73] are questioning the usefulness of this approach, since only rarely has standard fetal heart rate surveillance detected CHB in time for effective treatment. Evers et al. [74] suggested that utilizing antibody levels to categorize this population can enhance surveillance for CHB. Standard (weekly) screening is not cost-effective and leads to excessive resource utilization. 

In the past, the administration of fluorinated GCs, such as dexamethasone and betamethasone, were equally recommended. The rationale for their use was based on the idea that by crossing the placenta, they could act on the inflammatory component of cardiac damage. However, multiple studies [75,76] show that first-degree block does not worsen in fetuses with untreated mothers. Second-degree BAVs tend to regress or progress whether treated or untreated, whereas third-degree blocks never regress with steroid therapy [76]. A metanalysis of nine studies, conducted by Hoxha et al., analyzed 747 pregnancies in which fluorinated steroids did not demonstrate superiority over other treatments for patients with CHB [77]. In this study, the outcomes were live birth, prevention of incomplete CHB progression, pacemaker implantation, and extra-nodal disease. 

Thus, despite having minimal benefits, GCs can lead to a series of both fetal and maternal complications, including infections, osteoporosis, osteonecrosis, diabetes, IUGR, and oligohydramnios. It has also been noted that their preventive use has no impact on NL development. There have been no controlled studies assessing the effectiveness of plasmapheresis in cardiac NL. IVIGs were not demonstrated to prevent cardiac NL at a dose of 400 mg/kg [78]. In contrast, several studies [79,80,81] have demonstrated that the use of HCQ from the early GWs reduces the risk of NL, even in cases of NL in previous pregnancies. Considering the potential benefits, HCQ should be initiated before conception or as early as possible during the first trimester in women positive for anti-SSA/-SSB antibodies, particularly in those with a history of CHB. With no therapies proven effective for the occurrence of CHB, close monitoring is recommended. In cases of fetal distress, early delivery is indicated. It has also been observed that 2% of AV blocks may appear up to 1 month after birth; hence, these children should be monitored by an expert pediatrician in the early weeks of life. The only procedure that increases the survival of these infants is the implantation of a pacemaker. Indeed, 70% have undergone pacing by the age of 10 years [78]. 

#### 4.2.2. Preterm Birth and Cesarean Delivery

It is defined preterm birth when delivery occurs before the 37th GW. Lupus patients have a higher likelihood of experiencing preterm birth compared to the general population. In the United States, for instance, the percentage varies from 33% in pregnant women with SLE to 12% in the national average [82]. Several factors contribute to an increased risk of this complication. Inflammation, both local and systemic, leads to the release of cytokines, prostaglandins, and complement consumption [83]. Indeed, SLE patients often exhibit reduced levels of estradiol, which are directly proportional to placental health [34]. Risk is also related to the administration of oral PDN [84,85] and dysfunctions in the maternal or fetal hypothalamic–pituitary axis, which result in elevated placental corticotropin-releasing hormone levels, leading to increased synthesis of cortisol and prostaglandins [86]. The risk is amplified by disease activity and the occurrence of PE. Moroni et al. [87] identified risk predictors for preterm delivery which include SLEDAI, active nephritis, proteinuria (g/day), arterial hypertension, previous renal flares, quarterly change in a single unit in SLEDAI, and quarterly increase in daily proteinuria >1 g during pregnancy.

Indications for cesarean sections in lupus pregnancies are not different from those in the general population. Nevertheless, due to a higher incidence of complications such as PE or fetal distress in SLE patients compared to those in healthy women, cesarean sections are performed in 33% of SLE pregnancies. Nevertheless, a woman with lupus experiencing an uncomplicated pregnancy is not prevented from opting for a standard vaginal delivery.

#### 4.2.3. Fetal Growth Restriction and Small-for-Gestational-Age Infants

Fetal growth restriction (FGR) is defined as an estimated fetal weight or abdominal circumference <10th percentile for gestational age. It is a rare complication in healthy pregnancies; the risk increases in smokers or those with gestational hypertension. Several studies have compared birth weights between offspring of women with SLE and healthy women, matched for age and risk factors, revealing a higher risk of FGR and SGA infants (<5th percentile) in SLE pregnancies. The risk increased in cases of active disease or renal flares [40,88,89]. Furthermore, in APS, placental infarction leads to reduced nutritional supply to the fetus and consequent growth delay. A meta-analysis involving over 20,000 pregnant women revealed that when LDA was given before 16 weeks, there were significant dose-dependent reductions in the rates of FGR [90]. The likelihood of having a baby classified as SGA was reduced by 85% in patients who received HCQ during pregnancy [87]. 

## 5. Postpartum

Women with active disease in the six months before conception or during pregnancy are at a higher risk of experiencing a flare during the postpartum period (defined as six to eight weeks after birth). Therefore, close monitoring is recommended. Breastfeeding is possible, as almost all the treatments administered during pregnancy are compatible with lactation. In women with anti-Ro/SS-A and anti-La/SS-B antibodies, it is essential to provide counseling regarding subsequent pregnancies, especially about the use of preventive HCQ in future pregnancies. Additionally, it is important to remember that the prevalence of postpartum depression in Europe is estimated to be around 10% [91]. This condition should be considered in the differential diagnosis with neuropsychiatric SLE [92].

## 6. Controversies and Open Issues in Management of SLE Pregnancy

There are still a number of open and controversial issues in the field of pregnancy management. Among these, we have already mentioned the cost-effectiveness of intensive surveillance with fetal echocardiography in patients with positive anti-Ro/SSA and anti-La/SSB antibodies and no previous child with congenital heart block, which remains to be established [73,74]. The potential therapeutic use of fluorinated glucocorticoids in case of CHB development also remains debated [77]. Certainly, the use of HCQ from the beginning of pregnancy has led to a reduction in the number of NL cases. 

Regarding APS, many questions are still open. Although new classification criteria have been recently published, these are not diagnostic criteria. Therefore, there are gray zones, which are typically found in clinical practice. How should pregnant SLE patients be considered and treated if they are not fulfilling the APS classification criteria? If and how should only aPL-positive patients or patients with aPLs and recurrent miscarriages be treated? Particularly in the latter case, when dealing with losses before the 10th GW, it is challenging in clinical practice to rule out other causes of miscarriage.

In addition, a growing number of new molecules are being approved for the treatment of SLE, but only a small portion is considered safe to use during pregnancy. This basically stems from a single issue: the lack of trials that include pregnant women. An ethical debate about the risk–benefit balance in research on this special population remains open. Consequently, available data and thus clinical decisions are not based on randomized controlled trials but on retrospective observational studies, case series, and expert opinions. This results in a significant delay between the market availability and the use in pregnant women of drugs that are effective in maintaining disease control. Therefore, uncertainty remains about the use of some SLE therapies, such as BEL, during pregnancy. On the one hand, there is a lack of data regarding their safety profile. On the other hand, their discontinuation could lead to poor disease control, which in turn is a risk factor for adverse pregnancy events.

Furthermore, there are also no clear guidelines regarding the shared management between gynecologists and rheumatologists of pregnant patients with SLE. Both the EULAR [7] and ACR [2] recommendations emphasize the importance of a multidisciplinary approach in these patients, but methods and timing are not outlined. This leads to pregnancy management that varies by clinical center, leading to considerable heterogenicity in hospital approaches to SLE pregnancies.

All these open issues make clear that further efforts are needed by the scientific community to achieve standardized pregnancy management in rheumatologic diseases, resulting in more effective prevention of possible complications and prompt management of any adverse events.

## 7. Conclusions

Managing SLE during pregnancy requires a critical approach that balances maternal and fetal health. Preconception counseling, achieving disease control before pregnancy, vigilant disease monitoring, scrupulous medication assessment, and proactive management of potential complications are crucial. Nevertheless, despite advancements in medical care and a greater understanding of the pathophysiology of SLE pregnancy, adverse gestational events are still more frequent in women with SLE than in the general population. Therefore, ongoing and future studies are essential to further refine our understanding and management of SLE in pregnancy. By focusing on education, research, and multidisciplinary care, the impact of SLE on pregnancy can be reduced, leading to an improvement in the quality of life of affected women and their offspring.

## Figures and Tables

**Table 1:** EULAR checklist for preconception risk stratification in women with SLE.

**Relative Contraindications**
Active disease in the 6 months before conception Active lupus nephritis, especially if requiring teratogenic therapy Stroke or major thrombotic event in the previous 6 months Medications contraindicated during pregnancy Vaccination status: recommended vaccines not administered
**Absolute Contraindications**
Severe pulmonary hypertension Severe restrictive lung disease Severe renal insufficiency (eGFR < 30 mL/min/1.73 m^2^) Advanced heart failure Previous PE or HELLP syndrome despite appropriate treatment

**Table 2:** Guiding factors for delaying or contraindicating pregnancy.

**Risk Factors Related to SLE**
Disease activity in the 6–12 months before conception Lupus nephritis (active or historical) Serological activity (complement consumption, positive anti-dsDNA) Severe organ damage (kidney, heart, lungs, etc.)
**Risk Factors Associated with SLE**
Antiphospholipid antibodies Anti-SSA Ro/SSB antibodies Previous adverse pregnancy outcomes Previous thrombotic events

Legend: PE: preeclampsia; HELLP syndrome: hemolysis, elevated liver enzymes, and low platelets.

**Table 3:** Therapy management for SLE during pregnancy.

Rheumatologic Medications Safe Throughout Pregnancy	Rheumatologic Medications Usable in Pregnancy in Exceptional Circumstances	Rheumatologic Medications Contraindicated in Pregnancy
Hydroxychloroquine (HCQ) Azathioprine (AZA) Prednisone (PDN) < 7.5 mg/day Cyclosporine (CsA) Tacrolimus (TAC)	Cyclophosphamide (CYC) Belimumab Rituximab (RTX)	Methotrexate (MTX) Mycophenolate Mofetil (MMF) Leflunomide (LEF)

**Table 4:** Clinimetric indices of SLE activity during pregnancy.

Clinimetric Index	Difference from the Original Version	Assessment Time	Final Score	Flare Definition
LAI-P	Excluded: PGA, fatigue Added: fever, vasculitis, myositis CVA or TP: not scored in aPL+ pt Proteinuria: >500 mg/day or doubling if previous LN, with ↓ C’ o ↑ anti-dsDNA; rule out PE	2 weeks	0–2.6	Increase of 0.25 from the last evaluation
SLEPDAI	Rule out: PE, HELLP, E, infection, Bell’s palsy, placental problems, pregnancy TP Considered physiological factors during pregnancy: ↑ C’, bland knee effusions, ↑ lymphocytes, palmar erythema, hyperventilation due to progesterone and dyspnea due to enlarging uterus, postpartum alopecia	10 days	0–105	4–11: moderate activity; > 12: severe activity
m-SLAM	Excluded: weight loss, ESR, scale for miscellaneous disease manifestations Rule out: PE, E, FM, placental problems, chloasma Considered physiological factors during pregnancy: bland knee effusions, ↑ lymphocytes, palmar erythema, hemodilution, ↓ serum creatinine, hyperventilation due to progesterone, dyspnea due to enlarging uterus, postpartum alopecia	1 months	0-81	≥7: active disease
m-ECLAM	Proteinuria pathological if >500 mg/day after excluding PE Non-hemolytic anemia not considered ESR not considered	1–3 months	0–17.5	≥2: active disease
BILAG2004-P	Rule out: bland knee effusion, peripheral edema of pregnancy and mechanical pain, PE, E, HELLP, APS, melasma, chloasma Physiological during pregnancy: ↑ lymphocytes and neutrophils, ↓ serum creatinine, hemodilution, postpartum alopecia For DD LN-PE: added C’ levels and anti-dsDNA added; excluded hypertension	4 weeks	A–E	A: severe; B: moderate; C: mild stable disease; D: no disease activity; E: no current or previous disease activity

Legend: ↑: increase; ↓: reduction; aPL: antiphospholipid antibody; APS: antiphospholipid syndrome; C’: complement; CVA: cardiovascular accident; DD: differential diagnosis; E: eclampsia; ESR: erythrocyte sedimentation rate; FM: fibromyalgia; HELLP: hemolysis, elevated liver enzymes, and low platelets; LN: lupus nephritis; PE: preeclampsia; PGA: physician global assessment; Pt: patient; TP: thrombocytopenia.

**Table 5:** Differential diagnosis of NL and PE.

Biomarker	Lupus Nephirtis	Preeclampsia
Blood pressure	Normal or ↑	↑↑
Onset	At any time	>20th GW
Renal biopsy	May show signs of active nephritis	-
Serologic markers	↑ Anti-dsDNA antibodies ↓ Complement levels Normal uric acid Normal or ↓ PLTs	Not specific, may include ↑ Liver enzymes ↑ Uric acid ↓ PLTs
Urine analysis	Proteinuria ↑ Active sediment	Proteinuria ↑↑↑ Not active sediment
SLE or renal symptoms	Could be present and increasing	Stable
Angiogenic factors	↓ (e.g., angiopoietin-2)	↑ (e.g., soluble fms-like tyrosine kinase-1)
Anti-angiogenic factors	↑ (e.g., soluble endoglin)	-

Legend: ↑: increase; ↓: reduction, GW: gestational week; PLTs: platelets.

**Table 6:** ACR/EULAR definition of obstetric APS criteria.

Clinical Domains	Weight	Laboratory Domains	Weight
≥3 consecutive pre-fetal deaths (<10th GW) and/or ≥3 early fetal deaths (10th–16th GW)	1	Positive LAC (single, one time)	1
≥1 fetal death (16th–33rd GW) without severe PE or severe PI	1	Positive LAC (persistent)	5
Severe PE or PI with or without fetal death (<34th GW)	3	Moderate or high positive IgM aCL and/or aβ2GP	1
Severe PE and PI with or without fetal death (<34th GW)	4	Moderate positive IgG aCL and/or aβ2GP	4
		High positive IgG aCL or aβ2GP	5
		High positive IgG aCL and aβ2GP	7

Legend: a-β2GPI: anti-beta2-glycoprotein I antibodies; aCL: anticardiolipin antibodies GW: gestational week; LAC: lupus anticoagulant PE: preeclampsia; PI: placenta insufficiency.
